# Leveraging Microneedles for Raised Scar Management

**DOI:** 10.3390/polym17010108

**Published:** 2025-01-02

**Authors:** Zhengyun Jin, Young-Seong Kim, Joong Yeon Lim

**Affiliations:** Department of Mechanical, Robotics and Energy Engineering, Dongguk University, Jung-gu, Seoul 04620, Republic of Korea

**Keywords:** microneedle, hypertrophic scar, hydrogels, keloids, drug delivery

## Abstract

Disruption of the molecular pathways during physiological wound healing can lead to raised scar formation, characterized by rigid, thick scar tissue with associated symptoms of pain and pruritus. A key mechanical factor in raised scar development is excessive tension at the wound site. Recently, microneedles (MNs) have emerged as promising tools for scar management as they engage with scar tissue and provide them with mechanical off-loading from both internal and external sources. This review explores the mechanisms by which physical intervention of drug-free MNs alleviates mechanical tension on fibroblasts within scar tissue, thereby promoting tissue remodeling and reducing scar severity. Additionally, the role of MNs as an efficient cargo delivery system for the controlled and sustained release of a wide range of therapeutic agents into scar tissue is highlighted. By penetrating scar tissue, MNs facilitate controlled and sustained localized drug administration to modulate inflammation and fibroblastic cell growth. Finally, the remaining challenges and the future perspective of the field have been highlighted.

## 1. Introduction

Raised scars represent a significant dermatological and clinical challenge, with epidemiological data demonstrating substantial prevalence across various traumatic skin injuries. Approximately 15% of post-surgical incisions and over 70% of burn victims experience abnormal scar formation [1,2]. These abnormal scarring processes emerge from complex pathological wound healing mechanisms, driven by a multifaceted interplay of genetic predisposition and, more importantly, wound recovery dynamics. The etiology of raised scars is predominantly triggered by delayed healing processes and excessive extracellular matrix (ECM) deposition associated with various skin traumas, including acute burns, surgical incisions, inflammatory conditions, and bacterial infections [2,3].

Keloid and hypertrophic scars (HTSs) are the most common types of raised scars; they share many similarities in the underlying mechanism of formation and symptoms but exhibit distinct characteristics. HTSs are characterized by their rigid thickened structure confined within the original lesion boundaries, exhibiting erythematous, pruritic structures. However, keloid scars demonstrate a more progressive, excessive growth pattern that extends beyond the initial lesion’s boundaries, mostly sharing similar symptomatic profiles [4].

The management of raised scars has historically encompassed a diverse range of clinical approaches. Surgical interventions have traditionally been at the forefront, including sophisticated techniques such as Z-plasty, full-thickness skin grafting, and local flaps, especially in the case of Keloids [4,5]. These methods have been complemented by conservative treatments like corticosteroid injections and various physical therapies, including pressure treatment and radiation therapy [4]. However, these conventional approaches have significant limitations. Most treatments are expensive, inefficient, require specialized clinical facilities operated by trained professionals, cause significant patient discomfort (highly invasive), or take a long time to affect.

The initial application of MNs for skin can be traced back to the 1990s [6]. Afterward, MNs have garnered significant scientific interest as a minimally invasive, pain-free platform for therapeutic and diagnostic applications [7]. Clinical studies have demonstrated the high efficacy and safety of MN treatment, per se, in improving various dermatological conditions, including acne vulgaris, hyperpigmentation, hyperhidrosis, and skin rejuvenation [8,9,10].

Moreover, comprising micrometer-scale needles arranged in arrays, MNs enable the transdermal delivery of diverse therapeutic agents, including proteins, genes, growth factors, and small-molecule drugs [11]. The use of MNs as a drug delivery system offers several advantages over traditional intralesional injections, including controlled and sustained drug release, higher efficacy, and reduced patient discomfort [12,13]. Unlike conventional injections, which deliver drugs directly into scar tissues to achieve high local concentrations, MNs allow for more precise drug delivery, often enhancing therapeutic outcomes even with lower drug concentrations while minimizing pain. Frequent and high dosages of corticosteroids may cause side effects such as skin shrinkage and telangiectasia in over 60% of reported cases [14]. Additionally, the gentle penetration of the skin’s outer layers by MNs facilitates ease of self-administration, making them a convenient option for patients. This reduces the reliance on healthcare professionals for injections, potentially improving patient compliance and overall self-treatment effectiveness.

This review delves into the potential of MNs as a transformative tool for addressing raised scars. Herein, we present an overview of the mechanisms underlying the pathological formation of these scars, offering insights into their complex biological pathways. Subsequently, we elucidated how microneedle-based therapies strategically disrupt and counteract these underlying pathological processes, thereby facilitating scar regression and orchestrating a more optimal trajectory of skin regeneration and recovery.

## 2. Underlying Mechanism of Scar Development

**Figure 1:**
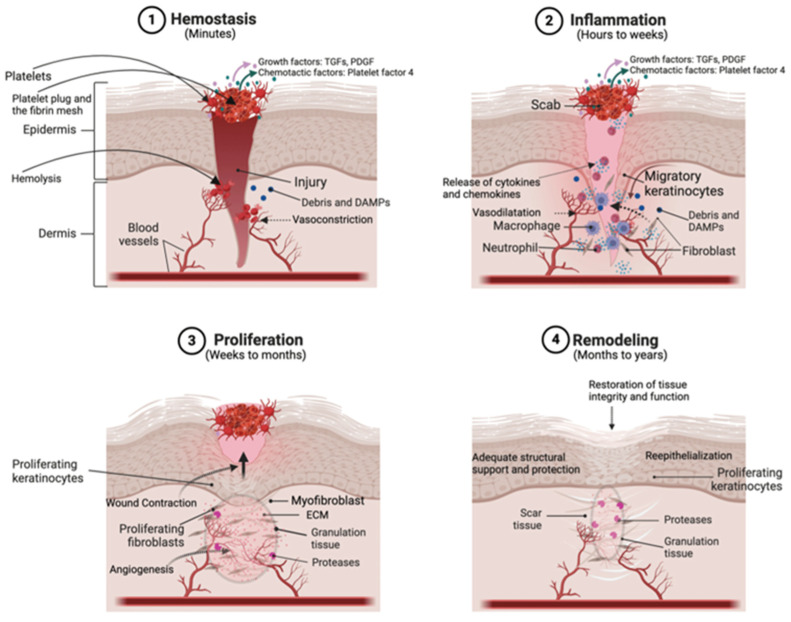
Schematic representation of different stages of wound healing [31]. Wound healing begins with hemostasis. Subsequently, damaged cells and platelet secretions recruit immune cells to the site, initiating the inflammatory phase. During this phase, immune cells release cytokines and chemokines to attract fibroblasts to the wound site and activate them. Activated fibroblasts differentiate into myofibroblasts, which deposit ECM components to form the structural framework for new tissue. Finally, in the remodeling phase, cellular activity significantly decreases, and myofibroblasts undergo programmed cell death (apoptosis).

Wound healing is a complex biological process characterized by four interconnected stages: (I) hemostasis, (II) inflammation, (III) proliferation, and (IV) remodeling (Figure 1). Disruptions in these stages can result in the formation of non-functional fibrotic tissue, commonly known as scars [15].

Following injury and successful hemostasis, the wound-healing cascade is initiated through cytokine release from platelets and damaged cells. These cytokines strategically recruit immune cells, including mast and myeloid cells, to the injury site [16]. Their primary object is to clear the pathogens, and cell debris, along with the secretion of a variety of cytokines, chemokines, and growth factors to facilitate tissue regeneration in the next steps of wound repair.

During the initial wound repair stage, myeloid cells play a critical role in tissue repair by secreting pro-inflammatory markers and chemokines. Specifically, chemokines like X-C motif chemokine ligand 14 (CXCL14) attract fibroblasts to the wound site, while transforming growth factor beta 1 (TGF-β1) promotes myofibroblast transition to accelerate wound repair [17]. In a groundbreaking study, Chen et al. [18] highlighted the pivotal role of myeloid cells in the early stages of wound repair, where they surpass fibroblasts in contributing to ECM production in the wound site. These immune cells release pro-inflammatory factors such as tumor necrosis factor (TNF) and CXCL14, which influence the inflammatory microenvironment and facilitate the recruitment, activation, and differentiation of fibroblasts into myofibroblasts [19]. Essentially, these immune cells set the stage for fibroblastic cells in the proliferation and remodeling phases of wound healing, but their number and activity naturally decline after the inflammation stage. In the proliferation stage, fibroblasts differentiate into myofibroblasts, marked by increased alpha smooth muscle actin (α-SMA) and Collagen I expression. These cells exhibit heightened contractility, proliferation, ECM production, and migration compared to fibroblasts, which are critical for wound closure and tissue repair.

Interestingly, studies have confirmed an elevated presence of macrophages and mast cells in HTSs compared to normal skin, indicating a deregulated inflammatory response in the wound [20]. Indeed, prolonged inflammation and subsequent sustained supplementation of fibroblastic cells with chemokines like TGF-b1, coupled with stiffened tissue due to excessive ECM accumulation, can create a positive feedback mechanism that extends the aberrant activity of myofibroblasts and increases ECM deposition and stiffness. Ideally, myofibroblasts undergo apoptosis once the wound heals, preventing excessive scarring [21]. Uncontrolled proliferation and hyperactivity of myofibroblasts as the main players of pathological skin repair are the primary contributors to tissue fibrosis.

Furthermore, mechanical stress affects the wound-healing process by modulating the behavior of cells within the ECM. Mechanical stretching of the ECM can activate latent TGF-β1, a critical factor for fibroblast and myofibroblast activity. This activation is facilitated by integrin-mediated force transmission, which causes a conformational change in the latent TGF-β1 complex, releasing its active form. Consistently high external mechanical tension, particularly in regions prone to cyclic mechanical stress such as joints, chest, nape, and shoulders, further promotes pathological fibrosis. This occurs through the direct regulation of fibroblastic cell behavior and through the release of latent TGF-β1 from the ECM [22,23,24]. The continuous presence of active TGF-β1 in the wound environment perpetuates fibroblast differentiation into myofibroblasts by activating the transcriptional co-activator Yes-associated protein (YAP). This process drives the nuclear localization and functional enhancement of Smad1 and Smad3, causing an abnormal increase in fibroblast-to-myofibroblast transition and promoting fibrosis formation [24,25,26].

Mechanistically, the external tension force on the wound area can directly activate fibroblasts and facilitate their differentiation [27]. Stretching forces on cells have been shown to activate mechanosensations through integrins, followed by focal adhesion formation, actomyosin polymerization, and nuclear YAP translocation [28,29]. As the main effector of the Hippo pathway, YAP significantly contributes to tissue fibrosis by upregulating ECM deposition gene expressions [30].

Conventional methods for managing HTSs and keloid scars typically involve a combination of medical and surgical strategies aimed at flattening, softening, or removing scar tissue. Several pharmacological treatments have been explored for HTS management, including intralesional corticosteroid injections (particularly triamcinolone acetonide) to reduce inflammation and collagen synthesis [32]; chemotherapeutic agents like 5-Fluorouracil and bleomycin, Tamoxifen, and Methotrexate to inhibit fibroblast proliferation and activity [33,34,35]; Mitomycin C for its anti-fibrotic properties; botulinum toxin A injections to reduce surrounding muscle tension [36]; verapamil as a calcium channel blocker to inhibit collagen production [37]; and Tranilast, an anti-allergic drug that inhibits collagen synthesis and inflammatory responses [38].

Silicone sheeting, another well-established technique, necessitates consistent and prolonged use, which can be inconvenient [39,40]. Cryotherapy, which employs extremely low temperatures to disrupt abnormal tissue growth, is occasionally paired with corticosteroid therapy to improve outcomes [41]. Surgical excision provides a more direct resolution but frequently requires supplementary measures, such as radiotherapy, silicone sheeting, or post-surgical steroid applications, to minimize recurrence [41].

Among the mentioned methods, many approaches, such as corticosteroid injections, silicone gels, and radiation, carry risks like skin damage or pigmentation changes, while advanced options like laser therapy are costly and less accessible in resource-limited settings. Additionally, high recurrence rates, especially with keloids, and variable outcomes influenced by scar size, location, and individual response further complicate treatment [42].

## 3. MNs for Raised Scare Management

Recently, the implication of MNs for scar management has gained a lot of attention as these platforms offer a unique blend of advantages. They are highly effective and affordable treatments for patients. Moreover, microneedle treatments are minimally invasive approaches that can often be self-administered, enhancing patient comfort and autonomy in scar management. Extensive research evidence has demonstrated the efficacy of MN patches in facilitating scarless wound repair, particularly as a preventative intervention for cases presenting high risks of raised scar formation [7,8]. However, MN-assisted strategies for raised scar management remain novel, and the treatment of keloids and HTSs continues to pose significant clinical challenges despite growing research interest. Table 1 summarizes the recent reports using MN for the management of raised scars.

### 3.1. Design, Material Selection, and Fabrication of MNs for Raised Scar Treatment

MN technology encompasses several distinct designs that revolutionize transdermal drug delivery through unique mechanisms and structures [48]. The fundamental types include solid MNs, which are simple rigid structures that create microchannels in the skin to enhance drug permeability [49]; coated MNs, which carry drug formulations directly on their surface for immediate release upon skin penetration [50]; and hollow MNs, which feature internal channels allowing for controlled liquid drug delivery similar to traditional hypodermic needles but at a microscale [51]. Dissolving MNs represents another innovative category, constructed from biocompatible polymers that completely break down within the skin while releasing their drug payload, eliminating biohazardous waste concerns [52].

**Figure 2:**
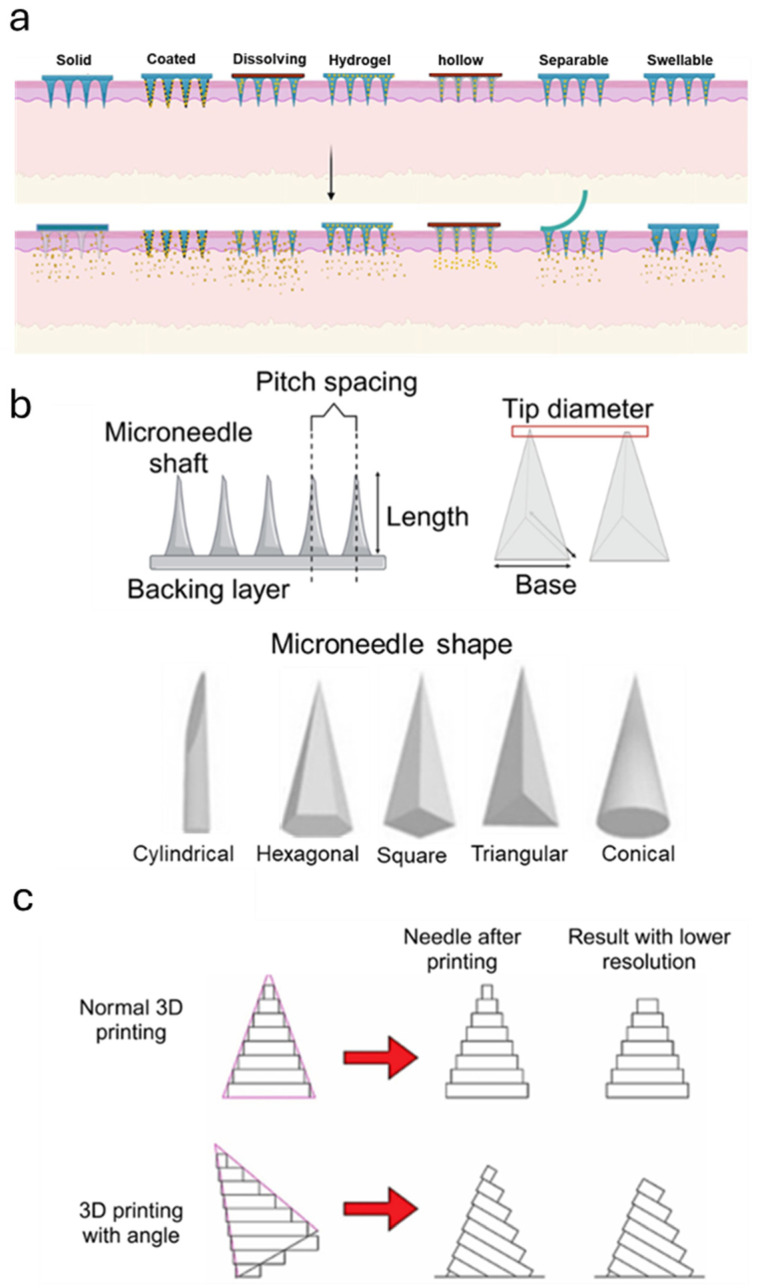
Schematic representation of microneedle patch design, material selection, and fabrication processes. (**a**) Different types of MN [72]. (**b**) Key design factors, including microneedle shape, tip diameter, base size, pitch spacing, length, and tip angle, significantly influence penetration efficiency and functionality. Various shapes, such as cylindrical, hexagonal, square, triangular, and conical, are depicted. (**c**) Comparison of standard 3D printing and angled 3D printing methods, highlighting the impact of resolution and needle angle [60].

Advanced MNs designs have emerged to address specific therapeutic needs and enhance drug delivery precision. These include hydrogel-forming or swellable MNs that expand upon contact with skin interstitial fluid, creating sustained-release pathways for drug delivery, and separable MNs that feature detachable tips that remain in the skin for prolonged drug release [53,54]. Additionally, smart MNs incorporate responsive materials that can modulate drug release based on specific triggers such as pH, temperature, or other environmental factors, enabling precise control over therapeutic delivery [55]. Each of these designs offers unique advantages for application in HTS management. Figure 2a demonstrates different types of MNs that can be applied to HTS management.

The design of MNs demands meticulous optimization of key factors, such as needle dimension (e.g., needle length, tip diameter, base geometry), tip angle, needle-to-needle spacing (pitch spacing), mechanical strength, and material properties (Figure 2b). These features must be engineered to ensure effective tissue penetration while minimizing pain, structural failure, and excessive trauma [9]. Particularly for raised scars, pain and pruritus (itching) are the most frequently reported symptoms [10].

Needle length is crucial for reaching the target tissue layer while ensuring patient comfort and safety. In raised scar treatments, the target cells are myofibroblasts located in the dermis, typically between 0.2 and 1.5 mm below the skin surface. Therefore, MNs must be long enough to penetrate this depth (not longer than 1000 µm) and effectively reach the desired therapeutic area without causing unnecessary discomfort [11,56,57]. MNs with longer lengths, such as 1500 µm, can lead to increased bleeding and inflammation [23].

The density and spacing of needles in MN patches influence treatment efficacy [58]. Too few needles may limit therapeutic effects, while overly dense arrays can increase trauma and inflammation. Moreover, mechanical strength is crucial for withstanding insertion forces, with design elements like thicker walls, optimized tip angles, and pyramidal bases enhancing durability [59]. A recent study on HTSs suggests that increasing microneedle density significantly enhances both the physical properties and therapeutic effectiveness [23]. Higher-density arrays in silk MNs showed greater hydrophobicity. This elevated hydrophobicity helped restrict cell migration, proliferation, and fibroblast-generated contractile stress in scar tissue. The therapeutic efficacy also improved with higher density, showing scar elevation reduction rates of approximately 3.2% (25 MNs/cm^2^, pitch spacing: 2300 µm), 13.2% (100 MNs/cm^2^, pitch spacing: 1070 µm), and 20.5% (225 MNs/cm^2^, pitch spacing: 690 µm). However, it is postulated that excessive array density beyond 20 × 20 MNs/cm^2^ would not facilitate better mechanical remodeling response, as it could lead to space compression and increased stress at needle tip contact sites [23].

The optimization of microneedle penetration capabilities is significantly influenced by adjusting the tilt angle during the manufacturing process. Recent exploration by Razzaghi and Akbari [60] demonstrated that a tilt angle of 45° applied across two axes proves to be the most effective configuration. This optimal angle arrangement results in notably sharper tips that can more efficiently penetrate the stratum corneum, reducing the required penetration force by 38% compared to non-tilted fabrication methods (Figure 2c). The enhanced tip sharpness, combined with improved mechanical stability achieved through this specific angular configuration, enables more precise and comfortable transdermal delivery while maintaining structural integrity for effective drug delivery and diagnostic applications.

The elastic modulus of raised scars has been reported to be around 150 kPa, which is up to around 10 times higher than the stiffness of healthy skin, typically around 17 kPa [61,62,63]. Variations in skin thickness across individuals and body regions also necessitate tailored designs to ensure effective and safe application. Therefore, material selection for MNs is critical, as it must provide the necessary stiffness and hardness to effectively penetrate these tougher scars, while still ensuring biocompatibility [64,65]. For example, silicon- and glass-based MNs suffer from brittleness, which limits their implications in scar treatment [66].

So far, a wide range of biocompatible materials, such as stainless steel, biopolymers (e.g., polyvinyl alcohol (PVA), polyvinylpyrrolidone (PVP), etc.), hydrogels (e.g., silk, gelatin methacrylate (GelMA), and hyaluronic acid methacrylate (HAMA), agarose, etc.), has been utilized for the preparation of MNs for skin conditions (Figure 2c) [67]. Silicon and nickel are materials that may exhibit low biocompatibility in microneedle applications due to specific challenges [40]. Silicon, while offering excellent mechanical strength and flexibility in design, is not FDA-approved as a biomaterial. Its brittle nature increases the risk of needle tips breaking during application, leaving fragments under the skin that could trigger undesirable immunogenic reactions or potentially migrate through blood vessels, causing complications such as arterial blockages [67,68]. Similarly, nickel-based MNs are associated with poor biocompatibility and are classified as a carcinogenic material [69,70]. Their use can lead to adverse effects such as skin irritation and toxicity, limiting their safety for biomedical applications unless coated with biocompatible layers like palladium or gold to mitigate exposure. These limitations highlight the need for careful material selection in microneedle fabrication.

Once the MNs have been properly designed and the appropriate material has been selected, the next step is to fabricate the MN patch. MN patches are fabricated using various techniques, including deep-reactive ion etching (DRIE), lithography, heat embossing, micromolding, 3D printing, and two-photon polymerization (TPP) [48,57,60]. Each method has distinct advantages and drawbacks regarding complexity, cost, and adaptability. For instance, DRIE allows precise control over MN size and shape but is expensive and requires cleanroom facilities, often resulting in fragile, non-biodegradable needles [71]. In contrast, heat embossing is cost-effective and produces robust MNs but is less suitable for hollow designs. Three-dimensional printing and TPP are innovative techniques that enable rapid prototyping and the creation of complex 3D structures without the need for master molds, making them ideal for applications like drug delivery and diagnostics. However, they may not achieve the high resolution required for certain porous MNs. Overall, while traditional methods like DRIE and lithography are widely used, emerging techniques like TPP and 3D printing are reshaping MN fabrication by enhancing design flexibility and application potential [56,71]. Micromolding is a widely used technique for manufacturing MNs, involving the use of a negative mold to form needles and load materials. It is compatible with a range of materials, including natural and synthetic polymers, ceramics, and hydrogels, and various curing methods such as solvent evaporation and photo-crosslinking are employed [56]. Noteworthily, it is unsuitable for processing materials like metals, silicon, and glass. This method offers advantages such as simplicity, low cost, and high reproducibility, and can create composite MNs with complex structures like multiregional or core–shell designs. However, challenges include issues with mold-filling due to surface tension, potential damage to fragile materials during demolding, and limitations in creating complex structures such as adhesive or hollow MNs.

### 3.2. MNs as a Physical Intervention

As previously mentioned, the ECM within scar tissue is subjected to significant tensile forces, primarily driven by the activity of highly contractile fibroblasts and external mechanical loading. MNs provide a unique physical intervention in this environment. As MNs penetrate the stiff, thickened ECM of scar tissue, they act as microscale reinforcements, redistributing mechanical stresses and alleviating localized strain concentrations [73]. This redistribution stabilizes the ECM and supports its structural integrity, functioning as a temporary microscaffold that enhances stress management. By promoting ECM alignment and delivering mechanical feedback, MNs mitigate excessive contraction and deformation, a hallmark of fibrotic scar progression [74].

**Figure 3:**
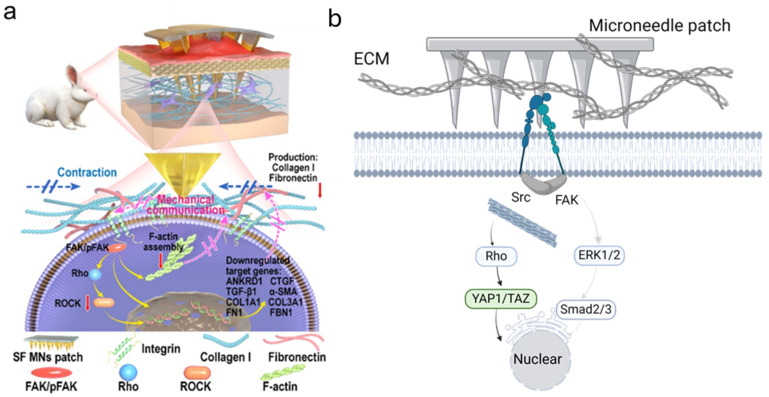
Schematic illustration of the physical intervention mechanism of microneedle patches. (**a**) Microneedle patches disrupt mechanical communication between cells and ECM by interfering with the integrin-FAK-mediated signaling pathway [23]. (**b**) Detailed representation of integrin-mediated mechanotransduction pathways, highlighting how microneedle-induced physical disruption affects mechanosensation. This intervention downregulates fibrotic gene transcription by inhibiting the integrin/FAK/ERK1-2 pathway, thereby reducing fibrotic responses and fibroproliferative activity (created with BioRender).

Research by Chengjie Xu’s group has provided compelling evidence of MNs’ efficacy in scar management [74,75]. In 2017, their study demonstrated that MNs, fabricated using FDA-approved liquid crystalline polymer (height: 500 μm, arranged in a 10 × 10 array with a density of 441 MNs/cm^2^), significantly reduced the viability of keloid fibroblasts, inducing up to 83.8% non-viable cells within 12 h of contact. This inhibitory effect was contact-dependent, as cells that did not directly interact with the MNs maintained normal growth and viability, confirming the absence of material cytotoxicity. Furthermore, MNs disrupted critical molecular pathways involved in scarring by downregulating markers such as α-SMA, Ki-67, and TGF-β1. In a human case study, a six-week application of MNs on an HTS alleviated erythema and pruritus, providing clinical evidence of their therapeutic potential (Figure 3a). Subsequent research in 2023 highlighted the impact of material composition on MN performance, demonstrating that different materials profoundly influence protein and gene expression patterns in a rabbit scar model. However, the underlying mechanisms of MNs’ therapeutic effects have not been thoroughly investigated. Future research should focus on elucidating the specific interactions between different MN materials and the scar microenvironment to emphasize the critical role of material selection in optimizing therapeutic outcomes.

In a related study, Zhang and coworkers explored the mechanistic basis of MN physical interventions in HTS treatment. Using a patch containing 225 MNs/cm^2^, they observed a dramatic reduction in fibroblast-induced collagen contraction from 50% to 4.1% compared to untreated samples. Mechanical restriction of collagen gels by MNs significantly decreased TGF-β1 and α-SMA protein levels, crucial mediators of fibrotic processes. RNA sequencing further revealed that MNs downregulated mechanosensitive genes, including ANKRD1, and fibroproliferative genes such as COL1A1, COL3A1, HIF1A, CTGF, and FN1, which are pivotal in ECM remodeling and focal adhesion pathways. Additionally, MN treatment diminished transcription of cell–ECM communication regulators, including FAK, pFAK, RhoA, Vinculin, and α-SMA, suggesting that MNs directly modulate mechanotransduction pathways (Figure 3b). These in vitro findings were corroborated by in vivo studies using a rabbit ear HTS model, where MNs disrupted the integrin/FAK signaling axis, thereby reducing the expression of fibrosis-associated genes and curbing scar formation. Complementing these findings, the Gurtner research group has extensively studied mechanosensitive pathways in fibrotic scars, particularly the integrin/FAK/ERK signaling cascade [18,76,77]. Their work demonstrated that targeting FAK signaling through pharmacological inhibition effectively prevented scar tissue formation in large animal models [78]. This convergence of evidence underscores the therapeutic potential of MNs as physical modulators of scar mechanobiology, paving the way for advanced scar management strategies.

### 3.3. Microneedles as Drug Carrier

While MNs provide a robust physical intervention by modulating mechanical stress and disrupting fibrotic pathways, incorporating pharmacological agents alongside them significantly enhances efficacy. This combination targets key biological processes, such as reducing inflammation, inhibiting fibroblast proliferation, and suppressing the fibroblast-to-myofibroblast transition, which is critical in scar formation. The sustained and micron-scale delivery of therapeutic agents through drug-releasing or dissolvable MN patches has garnered substantial interest in recent years. These systems enable localized and controlled delivery of pharmacological compounds directly into scar tissue. Various immunomodulatory drugs and inhibitors, including corticosteroids, anti-neoplastic drugs (such as 5-fluorouracil), mechanomodulatory inhibitors, and bioactive compounds from traditional Chinese medicine, have been explored to improve HTSs and keloids [79,80].

#### 3.3.1. Corticosteroids-Loaded MNs

TAC, along with hydrocortisone, dexamethasone, and betamethasone, represents a widely utilized class of corticosteroids in the therapeutic management of keloids and HTSs [81,82]. Corticosteroids exert their anti-inflammatory effects by binding to cytoplasmic glucocorticoid receptors, which regulate gene expression [83]. This process induces the production of lipocortin-1, an anti-inflammatory protein that suppresses phospholipase A2 activity, thereby reducing the level of pro-inflammatory mediators [84]. Additionally, corticosteroids induce lTh1 and Th17 responses, while promoting the differentiation of Th2 and regulatory T cells [85]. Furthermore, corticosteroids exhibit anti-fibroblast activity by blocking the progression of the cell cycle from the G0/G1 phase to the S phase and inducing fibroblast apoptosis. They also demonstrate anti-angiogenic effects by decreasing the mRNA levels of VEGF via the glucocorticoid receptor pathway [83,85]. Lastly, corticosteroids promote ECM remodeling by downregulating the synthesis of type I and III pro-collagen through modulation of TGF-β1 and bFGF expression. Additionally, they accelerate collagen degradation by reducing inhibitors like α1-antitrypsin and α2-macroglobulin [83].

TAC is the most prevalent corticosteroid that demonstrates both anti-inflammatory and anti-angiogenic properties through different molecular mechanisms [78,86,87]. At the molecular level, TAC downregulates critical fibrotic markers including TGF-β1, collagens, and integrins, which effectively inhibits fibroblast proliferation and adhesion [57]. This inhibition reduces differentiation into mesenchymal fibroblasts, a cellular subtype that drives excessive fibrosis. When paired with 5-fluorouracil (5-FU), TAC suppresses M2 macrophage activity, thereby diminishing their pro-fibrotic contributions [88]. The treatment also increases anti-inflammatory secretoglobulin expression, which disrupts fibrosis-promoting mechanisms and promotes a remodeling-prone microenvironment conducive to normal wound healing.

Chen et al. [83] have exploited layered hydrogel-based MNs to deliver compound betamethasone in a sustained manner to rabbit HTS models. To this end, they devised a dual cross-linked network structure that gives the MNs sufficient mechanical strength to penetrate scar tissue while regulating sustained drug release over 7 days. Interestingly, Chen and colleagues claimed that drug-free MNs with 100 MNs/cm^2^, height of 800 µM, and pitch spacing of 650 µm did not improve scar elevation index, which emphasizes the importance of MN patch design and material composition. However, the betamethasone-loaded MNs demonstrated significant therapeutic effects by decreasing the scar elevation index, reducing the ratio of collagen I/III, and lowering TGF-β1 protein expression in HTS models.

**Figure 4:**
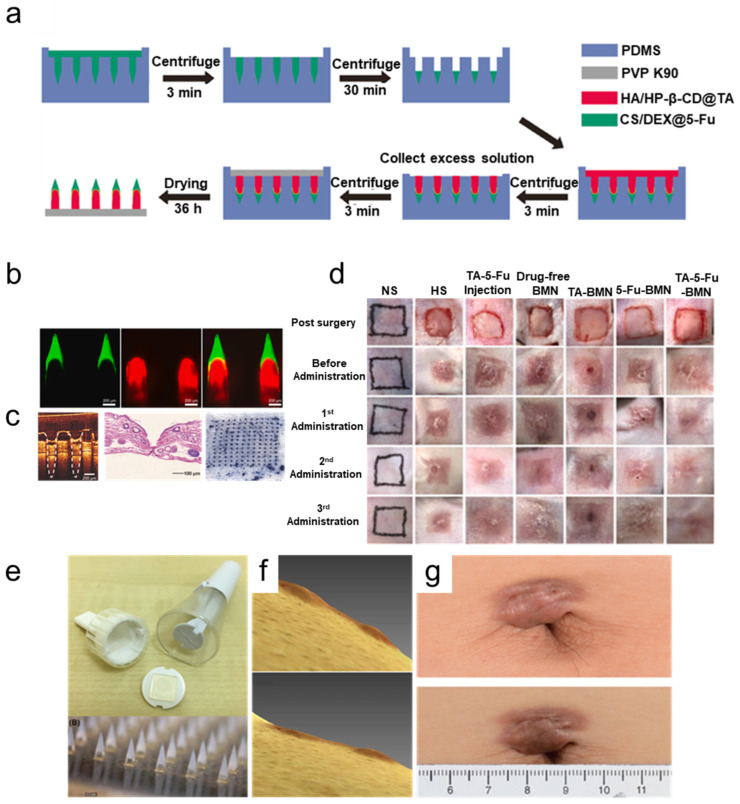
Corticosteroid-loaded MN patches for drug delivery. (**a**) Schematic representation of the bilayer MN patch fabrication process using the micromolding approach, illustrating the sequential loading of different drug formulations into the MN tips and shafts [46]. (**b**) Confocal microscopy images demonstrating the capability of MNs to compartmentalize different drugs between the tip and shaft regions for target delivery. (**c**) Photographic, optical coherence tomography (OCT), and histological images confirming effective MN penetration into the skin layers, showcasing their structural integrity and delivery efficiency. (**d**) Sequential photographs from the in vivo study highlighting the therapeutic efficacy of drug-loaded MN patches. Comparison of treated groups (drug-loaded MNs) with MNs alone and sham groups illustrates the superior performance and clinical potential of the drug-loaded patches in reducing symptoms. (**e**–**g**) The commercialized design of MNs for drug delivery [90]. (**e**) Commercially available soluble MNs for transdermal drug delivery. (**f**) Scanned 3D photos of the MNs-treated keloid. (**c**) Photographs of keloid scars before and after 6 weeks of applying the drug-loaded MNs show improvement in scar elevation index.

Yang et al. [46] reported an enhanced scar treatment using an engineered MN patch with a dual-layer structure that incorporated TAC and 5-Fu with distinct release kinetics. The needle tip layer was constructed using a blend of chitosan/dextran biomatrix specifically designed for controlled, sustained release of 5-Fu. The needle tail layer utilized hyaluronic acid as the carrier biomaterial and hydroxypropyl-beta-cyclodextrin as an inclusion compound to encapsulate the poorly water-soluble TAC, enabling its rapid release. This dual-layer architecture achieved a biphasic drug release profile that effectively reduced collagen deposition in scar tissue through the complementary actions of both therapeutic agents (Figure 4a–d).

Yang et al. [89] developed stimulus-responsive MNs to improve the delivery of bioavailable 5-FU for treating HTSs. These MNs, made of GelMA mixed with reactive oxygen species (ROS)-cleavable 5-FuA-Pep-MA pro-drug, release an active form of 5-FU in response to elevated ROS levels in scar tissue. Once inserted, the MN base layer detaches, leaving drug-loaded tips embedded for sustained action. The porous, hydrophilic GelMA structure absorbs interstitial fluid, enabling efficient drug exchange with the fibrotic microenvironment. The ROS-responsive pro-drug mechanism enables targeted drug release, with the peptide sequence undergoing cleavage in response to elevated ROS levels. This smart design, combined with the controlled biodegradation of the MNs over time, ensures that drug release is proportional to the pathological ROS levels, effectively addressing oxidative stress at the source. RNA sequencing confirmed that the MNs restored ECM enzyme activity (e.g., gelatinases and collagenases) to near-normal levels, addressing oxidative stress and promoting scar remodeling (Figure 4e–g).

In 2019, a clinical trial assessed the efficacy of TAC-loaded MNs for treating keloids [90]. The results revealed significant reductions in keloid volume, particularly at higher TAC dosages. The MNs dissolve painlessly within two minutes, enhancing patient comfort and allowing self-administration. While nearly half of the participants believed that traditional injection methods were more effective, a substantial majority preferred the MN approach due to its reduced pain and greater convenience. Notably, although the treatment effectively decreased keloid size, there was a tendency for keloids to regrow after treatment cessation, similar to outcomes observed with conventional therapies, indicating that multiple treatments with TAC-loaded MNs may be necessary to effectively minimize recurrence rates.

#### 3.3.2. Plant-Extract-Loaded MNs

Incorporating plant-derived phytotoxins, particularly those from traditional Chinese medicine, into MNs has emerged as a promising strategy due to their potent anti-inflammatory, immune-regulatory properties, and therapeutic potential for treating raised scars [80,91]. Extracts such as phenolic compounds (e.g., gallic acid), flavonoids (e.g., quercetin), naphthoquinone (e.g., shikonin), alkaloids (e.g., trigonelline), saponins (e.g., Panax notoginseng saponin), and oregano essential oil, are among the most commonly studied for addressing both HTSs and keloids [43,45,92,93,94,95,96,97]. Of note, since keloids are human-specific skin diseases, the lack of precise animal models has significantly restricted research on keloid treatment [98].

**Figure 5:**
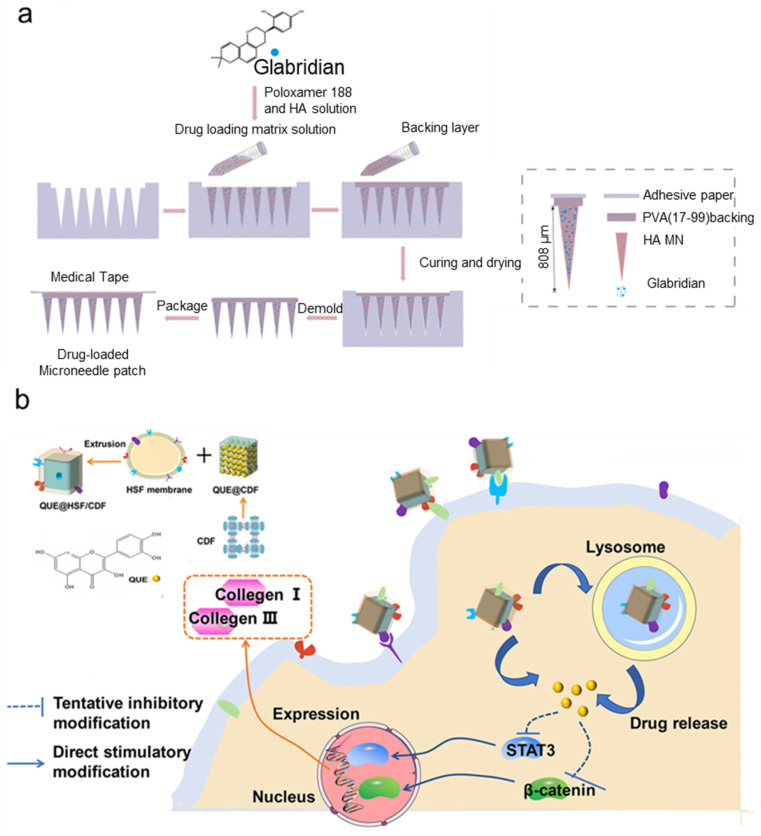
Schematic illustration of the fabrication process and therapeutic mechanism of plant-extract-loaded MNs for raised scar management. (**a**) Plant-extract-based MNs loaded with MNs for keloid treatment [99]. (**b**) Engineered MNs leverages cell membrane-cloaked QUE@ CDF particles to target collagen deposition in hypertrophic scars by modulating Wnt/β-catenin and JAK2/STAT3 signaling pathways [101].

For example, glabridin (Gla) has demonstrated significant therapeutic effects on uncontrolled hyperplasia that contributes to keloid formation, by inducing apoptosis in fibroblastic cells through the regulation of key signaling pathways, namely, PI3K/Akt and TGF-β1/SMAD. Guo et al. [99] have devised hyaluronic-acid-based MNs to enhance Gla delivery, improving its cumulative release rate by approximately 20% and facilitating deeper dermal penetration through microchannels (Figure 5a). Gla-MNs demonstrated superior efficacy compared to Gla alone by upregulating pro-apoptotic proteins (cleaved caspase-3, Bax) and downregulating anti-apoptotic protein Bcl-2. Additionally, both Gla and Gla-MNs inhibit PI3K/Akt phosphorylation and suppress TGF-β1/α-SMA signaling, reducing fibroblast hyperplasia and ECM production. These findings highlight Gla-MNs as a more effective strategy for mitigating scar hyperplasia and promoting scar reduction.

In another study, MNs have been engineered to deliver gallic acid (GA) and quercetin (QC) to combat keloid [100]. Qu-loaded amphiphilic gelatin nanoparticles were embedded in GA/gelatin-based MNs. This design enables rapid GA release upon MN dissolution, inhibits fibroblast proliferation by reducing cell viability within hours, and downregulates collagen synthesis, particularly type I and III collagen, and TGF-β1 expression. On the other hand, the sustained release of Qu from nanocarriers suppresses ROS over time and further inhibits fibrosis-related pathways by attenuating TGF-β1/Smad signaling and modulating PI3K/Akt activity. Together, GA and Qu reduce ECM overproduction, maintaining a balance between MMP-2 and TIMP-1, thereby preventing excessive ECM deposition.

Wu et al. [101] also reported using dissolvable MNs to leverage the therapeutic impact of Qu for treating HTSs. In this study, the diphenyl carbonate cross-linked cyclodextrin metal–organic framework (CDF) loaded with Qu was coated with HSF membranes and incorporated into dissolvable MNs made by polysaccharides for targeted localized therapy. This system effectively suppressed collagen I and III expression in HTSs by modulating the Wnt/β-catenin and JAK2/STAT3 signaling pathways (Figure 5b).

#### 3.3.3. MNs Loaded with Small Molecules

Another effective strategy for addressing raised scars involves targeting fibroblastic cells utilizing small molecules, particularly inhibitors, that can disrupt the molecular processes leading to excessive scar tissue formation.

The renin–angiotensin system, particularly angiotensin II and the AT1 receptor, plays a crucial role in HTS formation by promoting fibroblast activity, collagen deposition, cell proliferation, and migration [102,103]. Losartan, a potent angiotensin II receptor blocker, works by selectively blocking the AT1 receptor, thereby inhibiting the pro-fibrotic and pro-inflammatory actions of angiotensin II [104]. In 2023, Huang et al. [105] developed customized dissolving MNs specifically for efficient and targeted delivery of losartan into HTSs. The MNs were composed of a gelatin and starch mixture, chosen for their biocompatibility, dissolvability, and adequate mechanical strength to ensure successful scar tissue penetration. Losartan-loaded MNs inhibited fibroblast proliferation and migration while reducing collagen deposition due to downregulation in pathways mediated by TGF-β1, as well as IL-6, a pro-inflammatory cytokine associated with excessive collagen production.

While strategies aimed at inhibiting the formation and development of raised scars have been explored, inducing cell death through the administration of small molecules presents an alternative promising method to tackle these abnormal scarring responses [106,107]. Dupilumab and Bleomycin are among the most efficient drugs [79,107]. Bleomycin is one of the vastly utilized small molecules in the treatment of raised scars, outperforming other methods like TAC, 5-FU, and their combinations in reducing scar size and recurrence rates [107]. Bleomycin is primarily an anticancer drug that works by inducing DNA damage and apoptosis in the cells [106]. Basically, it interacts with iron ions to form a complex that generates ROS, causing oxidative DNA strand breaks. This mechanism is particularly effective against rapidly dividing cells such as activated fibroblastic cells [108,109].

### 3.4. Combinative Strategies Based on MNs

Photodynamic therapy (PDT) has emerged as a quite-new technique for raised scar ablation. This method involves activating photosensitizers with visible light to produce ROS, which targets and eliminates HTS fibroblasts and myofibroblasts [110]. Briefly, laser photons excite photosensitizer molecules, elevating them to an excited state. In this state, the photosensitizer transfers energy to nearby oxygen molecules, producing highly reactive and toxic ROS. These ROS disrupt cellular membranes and other critical structures, ultimately inducing cell death [110,111,112]. Importantly, effective PDT requires the precise delivery of a potent photosensitizer to the scar site. Therefore, the MN patch offers a precise delivery system, enhancing PDT performance by ensuring proper photosensitizer concentration at the target site.

**Figure 6:**
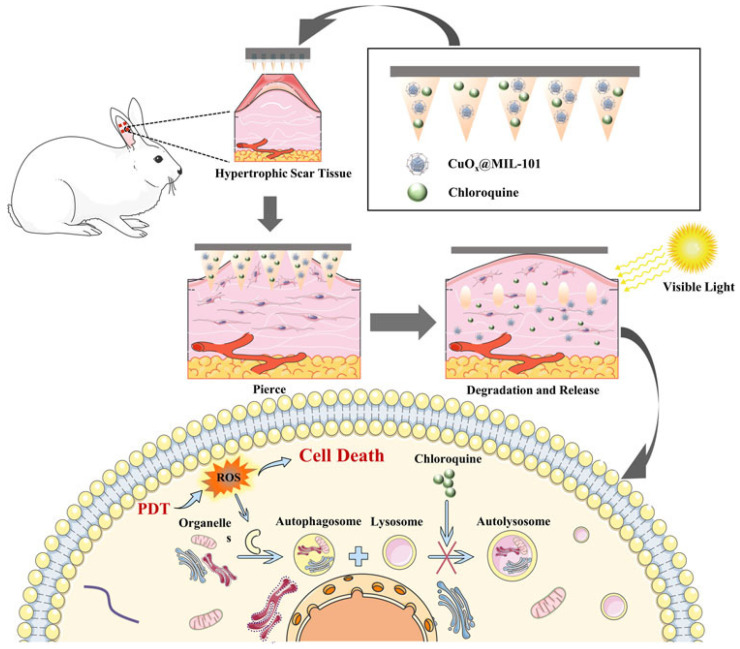
Schematic illustration of CuO₂@MIL-101 and chloroquine-loaded MNs for a synergistic strategy combining PDT and autophagy inhibition to treat HTSs [113]. The dissolving MNs penetrate the hypertrophic scar tissue, enabling localized delivery of CuO₂@MIL-101 and chloroquine. Upon visible light irradiation to nanoparticles, destructive ROS are generated through PDT, leading to cell death. Concurrently, chloroquine disrupts autophagy by inhibiting lysosome–autophagosome fusion, enhancing the therapeutic effect.

Recently, Chen and colleagues [113] exploited a combination of photosensitizer CuO-dopped Cr-based metal–organic frameworks (called CuOx@MIL-101) and autophagy inhibitor chloroquine for ablation of HTSs in a rabbit model. Their results showed that this combination modulates autophagy, where initial protective mechanisms in cells can be disrupted with chloroquine to enhance efficacy. Additionally, it reduces inflammation and ECM deposition, aiding tissue remodeling. The success of PDT hinges on efficient photosensitizer delivery and precise light activation, with MNs emerging as an effective method to penetrate and deliver these agents into raised scar tissues (See Figure 6).

MNs are powerful tools that enable localized administration of therapeutic agents deep into the skin layers. However, there may be additional obstacles hindering efficient drug mobility and penetration through the skin barrier [114]. Sonophoresis is a useful method that uses ultrasound to create microbubbles that induce cavitation, forming temporary pores in the skin and increasing permeability [115]. The heat generated disrupts the lipid structure of the stratum corneum, while mechanical vibrations break down cellular tight junctions, all of which enhance the diffusion of drugs through the skin. This combination improves the transdermal delivery of drugs, even large molecules. On the ex vivo samples of normal scars and keloids, Yang et al. [114] studied the penetrating ability of the TAC drug using sonophoresis-assisted MNs. They found that 600 μm MNs penetrated the dermal layer in 90% of normal scars but only 38% of keloids due to their thicker epidermis. Combining MN pretreatment with low-frequency ultrasound (sonophoresis) significantly enhanced the delivery of TAC into both scar types compared to MNs alone. Ultrasound vibrations likely drove drugs deeper through MN-created micropores, improving drug penetration for potential keloid treatment.

Electroporation is a technique that transiently disrupts the lipid bilayer structures in the stratum corneum by applying short, high-voltage electric pulses [116]. This creates aqueous pathways within the lipid matrix, facilitating the transdermal delivery of hydrophilic molecules. Additionally, the applied electric field generates a localized electrokinetic driving force that further promotes the movement of therapeutic agents across the skin barrier. Consequently, electroporation has demonstrated significant enhancement in the percutaneous absorption of drugs and biomolecules [115]. Calcium electroporation involves the use of high calcium concentrations and electric pulses to disrupt keloid cell membranes [117]. This process facilitates calcium entry into cells and induces cytotoxic effects that reduce keloid growth. It provides a non-mutagenic and minimally invasive alternative to chemotherapy. Challenges include the need to optimize dosing and address recurrence while validating efficacy through larger clinical trials. This method holds potential as a safe and effective keloid treatment. The administration of TAC in a combinative approach involving MNs and electroporation has been proposed for keloid in clinical settings [118].

A combination of MNs and fractional radiofrequency (RF) is an innovative approach for treating various pathological skin scars, including raised scars [65,119,120]. This method, called fractional microneedle radiofrequency (FMR), involves the insertion of MNs into the scar tissue, where RF energy activates a dual mechanism for addressing scar tissue. It combines mechanical disruption with the generation of heat through an alternating electric current within the dermis, causing thermal damage that denatures collagen and stimulates subsequent neo-collagenases [121,122]. A recent report by Tawfic et al. [119] revealed that FMR has demonstrated high efficacy in improving scar appearance and pliability. Indeed, FMR stimulates fibroblasts to release growth factors like VEGF and PDGF, which promote organized collagen and elastin deposition. Simultaneously, it downregulates TGFβ1, a pivotal driver of fibrosis, thereby limiting excessive scar tissue formation. The resulting scar exhibits improved structural integrity, reduced rigidity, and minimal surface disruption compared to ablative fractional lasers. FMR’s targeted dermal remodeling and minimal epidermal injury contribute to its favorable safety profile and reduced risk of adverse effects like post-inflammatory hyperpigmentation.

## 4. Challenges and Future Perspective

Current treatments for HTSs and keloids, such as silicone sheeting and corticosteroid injections, face limitations, including high recurrence rates for keloids, regulatory barriers, cost constraints, and inconsistent efficacy [41]. These issues, combined with side effects like skin irritation and discoloration, reduce accessibility and patient satisfaction [123]. Consequently, minimally invasive methods like MNs have garnered attention. However, challenges remain in realizing the full potential of MN-based approaches for scar management.

The successful implementation of MN treatments faces several technical challenges that require careful consideration. One of the primary concerns is maintaining uniform insertion depth across applications, while simultaneously ensuring that the treatment delivers its intended therapeutic effects. Additionally, there is a critical requirement for comprehensive clinical validation studies to establish standardized protocols that can optimize therapeutic outcomes. These protocols must be developed through meticulous experimental design to ensure reliable and reproducible results. The emphasis on standardization and careful methodology is particularly important in this context, as it directly impacts the consistency and reliability of treatment outcomes.

From a drug delivery perspective, MNs face limitations in drug loading capacity and permeability through dense scar tissue. Conventional MNs often fail to accommodate the high drug concentrations needed for effective treatment. Innovations like porous nanocarriers, such as metal–organic frameworks (MOFs), have been explored to enhance drug encapsulation and sustained release [124]. However, incorporating nanoparticles can compromise the mechanical strength of MNs, necessitating reinforcement through optimized materials [125,126].

The stiffness and thickness of scar tissue further limit drug mobility, reducing therapeutic efficacy. Combining MNs with complementary technologies like sonophoresis, iontophoresis, or electroporation has been proposed to improve drug penetration and outcomes [127,128,129,130]. These approaches, while promising, require refinement to ensure safety, efficacy, and affordability for broader clinical use.

Exosomes, nanoscale extracellular vesicles containing bioactive molecules like RNA and proteins, are promising tools for scar treatment due to their ability to mediate intercellular communication and target pathological microenvironments. Specifically, exosomes derived from adipose mesenchymal stem cells (ADMSC-Exos) demonstrate significant therapeutic potential in scar treatment through their ability to modulate key fibrotic pathways [131]. These vesicles function by inhibiting critical signaling cascades, particularly the TGF-β/Smad and Notch-1 pathways, which are instrumental in regulating the transformation of fibroblasts to myofibroblasts and subsequent excessive ECM production [132]. Additionally, ADMSC-Exos help maintain ECM homeostasis by modulating the balance between matrix metalloproteinases (MMPs) and their tissue inhibitors of metalloproteinases (TIMPs) [133]. Exosome-loaded MNs offer a superior delivery platform, overcoming challenges like low extraction yield and rapid clearance of exosomes in circulation. Exosome-based therapy holds tremendous promise for raised scar management, and further research is essential to fully unlock its potential, particularly when combined with MN technology.

## 5. Summary

MNs represent a versatile therapeutic approach for managing HTSs and keloids through both physical intervention and drug delivery mechanisms. As a physical intervention, MNs penetrate the rigid ECM of scar tissue, redistributing mechanical stresses and providing microscale reinforcement that stabilizes the ECM. MNs can significantly reduce keloid fibroblast viability and disrupt critical molecular pathways involved in scarring by downregulating markers such as α-SMA, Ki-67, and TGF-β1. When used as drug carriers, MNs can effectively deliver various therapeutic agents, including corticosteroids, plant extracts, and small molecules, directly into the scar tissue to modulate immune responses and inflammation, regulate fibroblast activity, and balance collagen production. The effectiveness of MNs can be further enhanced by combining them with other therapeutic strategies, such as photodynamic therapy, sonophoresis, and electroporation, offering promising avenues for improved scar management.

## Figures and Tables

**Table 1:** Recent advancements in MNs for the treatment of HTSs and keloids.

MN Type	Material Composition	Therapeutic Agent	Scar Type	Outcome	Ref.
Layered dissolving MNs	Sodium carboxymethyl cellulose	Asiaticoside (AS), ginsenoside Rb1, and L-carnosine	HTSs	Enhanced therapeutic effects comparable to triamcinolone acetonide (TAC) injections. Improved drug delivery through skin barrier.	[43]
Dissolving MNs (DMNs)	Dextran-Polyethyleneimine (PEI)	TAC	HTSs and Keloid	TAC-DMNs showed significant reduction in HTSs, while pristine DMN and TAC-DMNs showed no impacts on keloids.	[44]
DMNs	Mucin	Oregano essential oil	HTSs	Leveraged anti-inflammatory properties of oregano essential oil. Effective delivery through microneedle system.	[45]
Bilayer dissolving MNs (BMNs)	Chitosan and Dextran for tips	TAC and 5-Fluorouraci	HTSs	TA-5-Fu-BMN, with its ability to reduce fibroblast proliferation, collagen deposition, and scar elevation while downregulating Collagen I and TGF-β1.	[46]
DMNs	Hyaluronic acid incorporated with Hydroxypropyl-β-cyclodextrin for tips	miR-141-3p-functionalized exosomes	HTSs	miR-141OE-Exos@DMNs reduces scar thickness, modulates the TGF-β2/Smad pathway, improves collagen fiber organization, and enhances fibroblast distribution.	[47]
